# Multiprotein Adsorption from Human Serum at Gold and Oxidized Iron Surfaces Studied by Atomic Force Microscopy and Polarization-Modulation Infrared Reflection Absorption Spectroscopy

**DOI:** 10.3390/molecules28166060

**Published:** 2023-08-15

**Authors:** Jingyuan Huang, Yunshu Qiu, Felix Lücke, Jiangling Su, Guido Grundmeier, Adrian Keller

**Affiliations:** Technical and Macromolecular Chemistry, Paderborn University, Warburger Str. 100, 33098 Paderborn, Germany; hjy9301@mail.upb.de (J.H.); yunshuqiu@yahoo.com (Y.Q.); fluecke98@gmx.de (F.L.); sujl@mail.uni-paderborn.de (J.S.); guido.grundmeier@uni-paderborn.de (G.G.)

**Keywords:** biointerfaces, protein adsorption, serum, AFM, PM-IRRAS

## Abstract

Multiprotein adsorption from complex body fluids represents a highly important and complicated phenomenon in medicine. In this work, multiprotein adsorption from diluted human serum at gold and oxidized iron surfaces is investigated at different serum concentrations and pH values. Adsorption-induced changes in surface topography and the total amount of adsorbed proteins are quantified by atomic force microscopy (AFM) and polarization-modulation infrared reflection absorption spectroscopy (PM-IRRAS), respectively. For both surfaces, stronger protein adsorption is observed at pH 6 compared to pH 7 and pH 8. PM-IRRAS furthermore provides some qualitative insights into the pH-dependent alterations in the composition of the adsorbed multiprotein films. Changes in the amide II/amide I band area ratio and in particular side-chain IR absorption suggest that the increased adsorption at pH 6 is accompanied by a change in protein film composition. Presumably, this is mostly driven by the adsorption of human serum albumin, which at pH 6 adsorbs more readily and thereby replaces other proteins with lower surface affinities in the resulting multiprotein film.

## 1. Introduction

When a biomaterial is brought into the body, it interacts with complex biological fluids that are composed of many highly diverse molecular species, ranging from inorganic ions to carbohydrates to proteins [1]. It is in particular the proteinaceous fraction that plays the dominant role in deciding the biological fate of implanted biomaterials [2]. Consequently, numerous studies have investigated protein adsorption at the surfaces of relevant materials in vitro with the aim of understanding and ultimately controlling the involved mechanisms and interactions [3,4,5,6,7,8,9,10,11,12,13]. The vast majority of such in vitro studies focused on the adsorption of one or a few selected proteins from single-component solutions. Body fluids, however, are composed of a multitude of protein species, with the total body-fluid proteome comprising more than 15,000 different proteins [14].

Among the different body fluids, blood plasma is not only the most relevant with regard to implants but also the most complex, with over 12,000 different plasma proteins [14]. Protein adsorption from such complex multi-component solutions is highly competitive and leads to the formation of a dynamic adsorbate film with changing composition that is determined by the individual concentrations and surface affinities of all the proteins in solution [15]. Investigating multiprotein adsorption under such conditions is experimentally challenging since established techniques such as quartz crystal microbalance, ellipsometry, or surface plasmon resonance only enable the quantification of the total amount of adsorbed proteins [16,17,18,19,20,21], while only a few techniques are able to provide information about the composition of the adsorbed protein films. Gel electrophoresis, for instance, enables the detailed identification of adsorbed proteins based on their electrophoretic mobility [22]. For this, however, the adsorbed proteins first need to be recovered from the surface. In contrast, MALDI-MS allows for the discrimination of proteins within the adsorbate film but not in a quantitative way [23]. In addition, assigning the detected masses to known plasma proteins may be rather challenging in some cases [23]. Also, immunolabeling of adsorbed proteins can been used to detect pre-selected plasma proteins in the adsorbate film [18,21], but the results are very sensitive toward multilayer formation, differences in protein packing, and protein denaturation, and thus are often rather ambiguous [3,24].

In this work, we evaluate the potential of atomic force microscopy (AFM) and polarization-modulation infrared reflection absorption spectroscopy (PM-IRRAS) to provide insights into multiprotein adsorption from diluted human serum. The most abundant proteins in human serum are listed in Table 1. AFM is used to obtain information about changes in surface topography due to the adsorbed multiprotein film, while PM-IRRAS enables the assessment of the total adsorbed protein amount and additionally allows us to qualitatively detect changes in protein film composition. In this way, multiprotein adsorption at the surfaces of gold and iron thin films is studied ex situ at different serum concentrations (1% and 10%) and pH values ranging from pH 6 to pH 8. This rather modest variation around neutral pH was chosen in order to minimize the contribution of protein denaturation in bulk solution. Gold was selected as a chemically inert model surface, whereas iron is chemically reactive and prone to corrosion and dissolution in physiological electrolytes, a phenomenon strongly influenced by the presence of proteins [25]. Our results show increased protein adsorption and a corresponding change in the composition of the adsorbed multiprotein film at both surfaces at pH 6 compared to pH 7 and pH 8. We assume that this is due to increased adsorption of human serum albumin (HSA) at the lower pH value.

## 2. Results and Discussion

### 2.1. AFM Results

**Figure 1:**
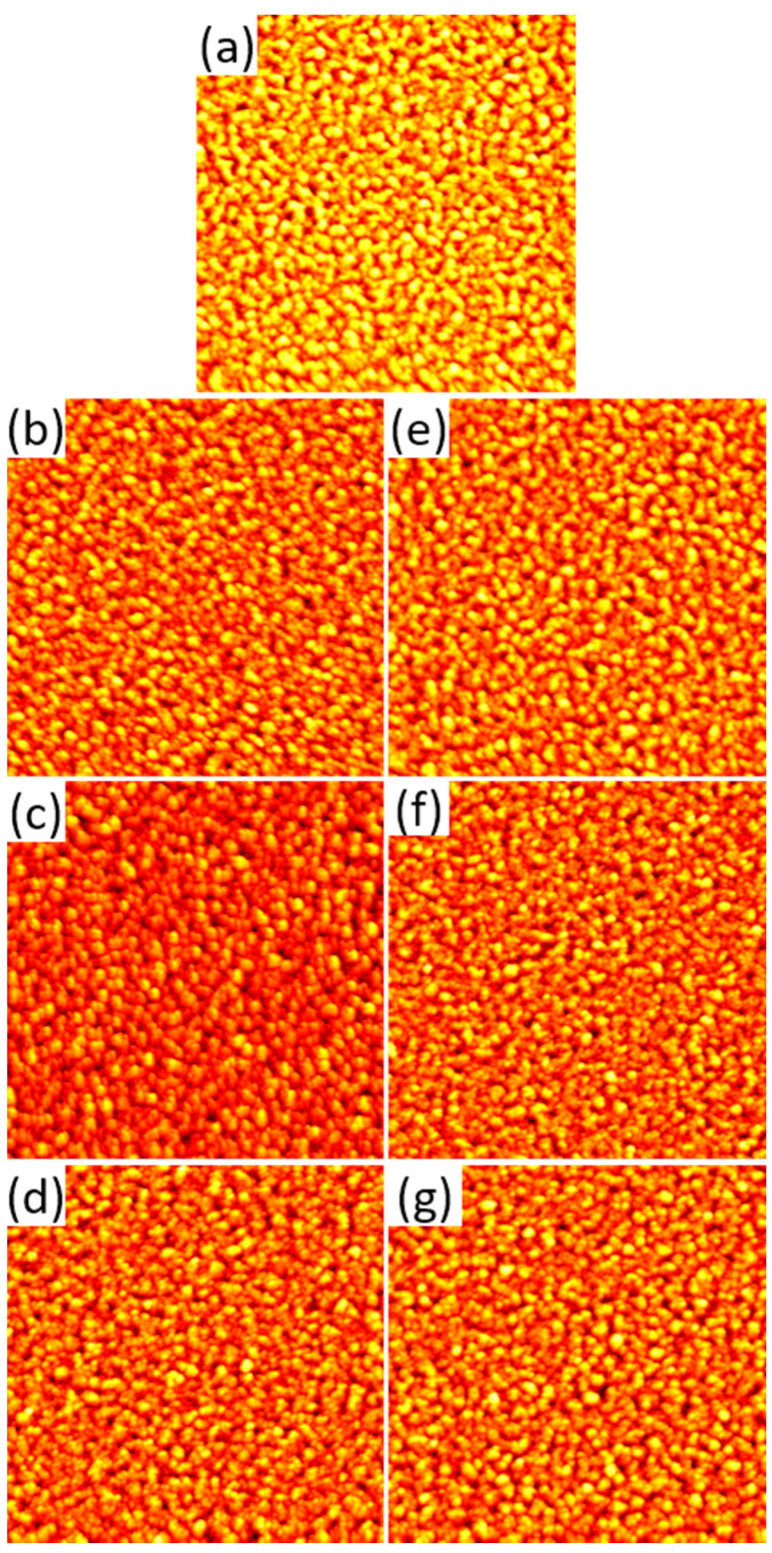
AFM images of the gold films before (**a**) and after exposure to 1% (**b**–**d**) and 10% (**e**–**g**) human serum at pH 6 (**b**,**e**), 7 (**c**,**f**), and 8 (**d**,**g**), respectively. The images have a size and height scale of 3 × 3 µm^2^ and 35 nm, respectively.

Before serum exposure, the surface topographies of the films were characterized by AFM. As can be seen in Figure 1a, the gold surface has a grainy appearance, resulting from the polycrystalline texture of the film. From the AFM images, a root-mean-square (RMS) surface roughness of the gold films of *S*_q_ = 4.39 ± 0.20 nm is obtained. After serum exposure under the different conditions, the topographies of the gold films differ only slightly from the as-prepared sample. As can be seen in the AFM images in Figure 1b–g, the overall grainy morphology is largely preserved with no apparent changes. This indicates the formation of a rather homogeneous protein film at the gold surface. However, for all conditions, the overall height contrast appears to be lower than for the as-prepared substrate in Figure 1a. Such a smoothing of the apparent surface topography may occur during the adsorption of relatively small and soft proteins such as serum albumin at comparably rough surfaces as a result of preferential adsorption of the proteins in topographic depressions, which allows them to maximize their contact area with the surface [3].

**Figure 2:**
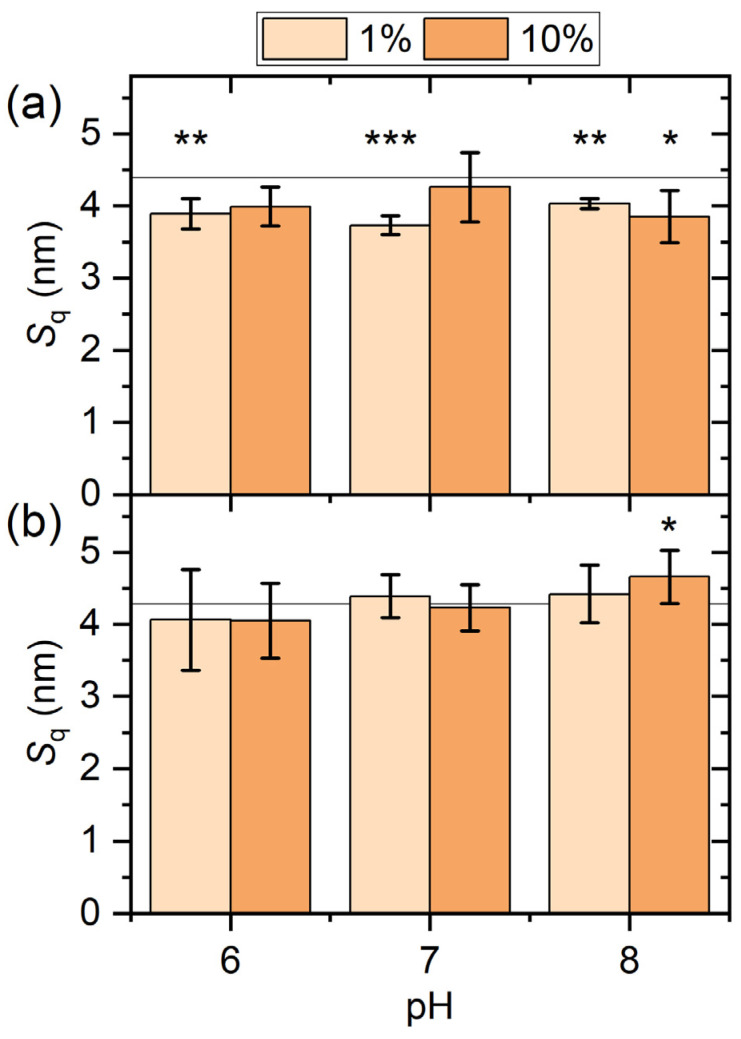
RMS surface roughness values *S*_q_ of the gold (**a**) and iron (**b**) films after exposure to human serum at different concentrations and pH values. The horizontal lines indicate the average *S*_q_ values of the as-prepared substrates prior to serum exposure. Values represent averages of four to eight AFM images recorded at different positions on the surfaces of two to four identically treated samples. Error bars indicate standard deviations. Significances (two-tailed distribution, homoscedastic) are given with respect to the as-prepared substrates and indicated as * (*p* < 0.05), ** (*p* < 0.01), and *** (*p* < 0.001).

The adsorption-induced changes in the topography of the gold surfaces are quantified in Figure 2a, which shows the RMS surface roughness values after serum exposure. In line with the qualitative observations discussed above, the RMS roughness values of the serum-exposed gold surfaces are significantly lower than that of the as-prepared substrate. The only exceptions are observed for the 10% serum at pH 6 and pH 7, which do not yield significant differences. As another interesting observation, the *S*_q_ values obtained under the different exposure conditions are very similar and do not seem to depend on pH or serum concentration.

**Figure 3:**
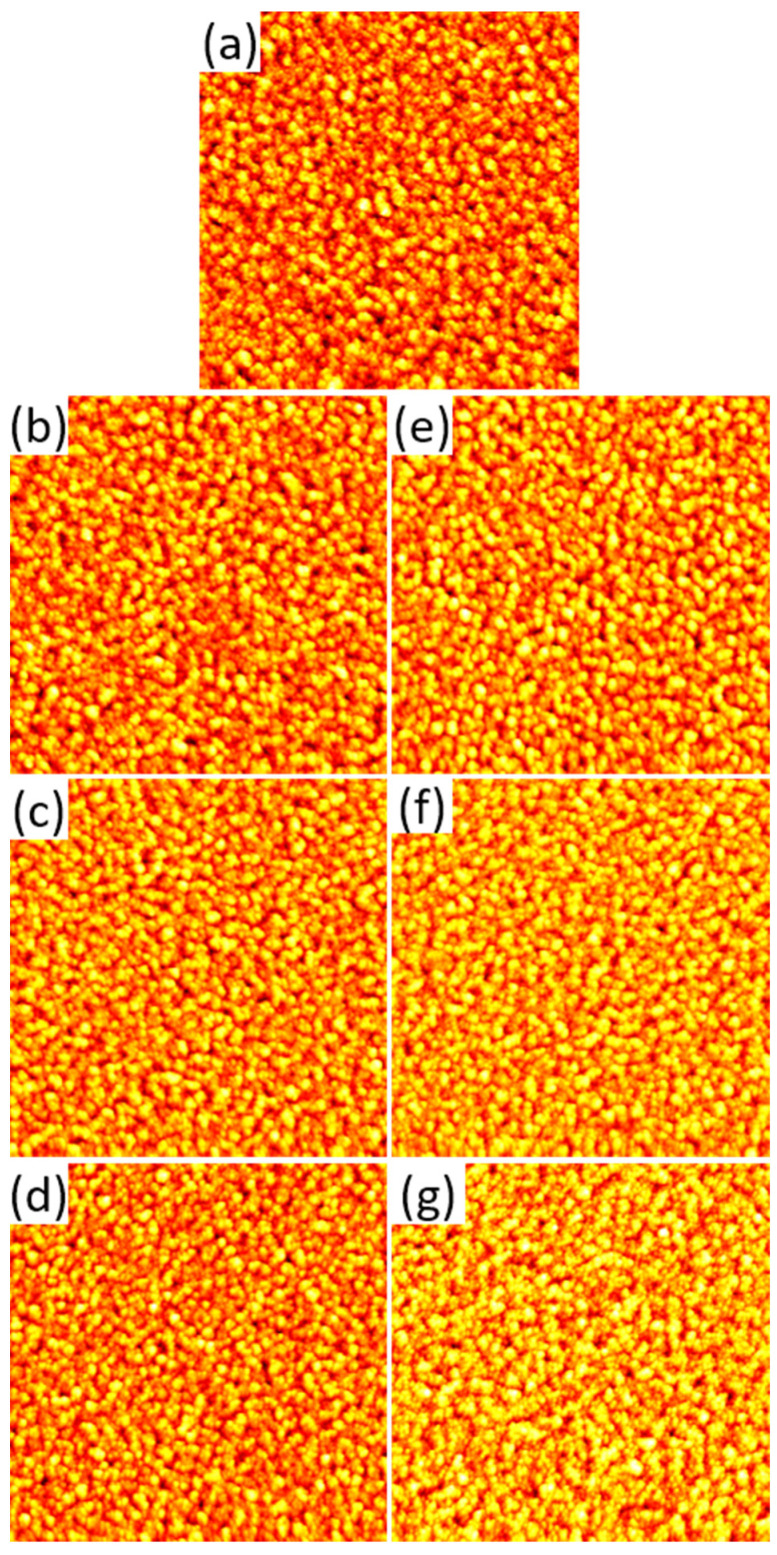
AFM images of the iron films before (**a**) and after exposure to 1% (**b**–**d**) and 10% (**e**–**g**) human serum at pH 6 (**b**,**e**), 7 (**c**,**f**), and 8 (**d**,**g**), respectively. The images have a size and height scale of 3 × 3 µm^2^ and 35 nm, respectively.

For the reactive iron surface the situation is similar, with the overall grainy surface morphology being largely preserved (see Figure 3). However, in contrast to the gold films the RMS surface roughness (*S*_q_ = 4.28 ± 0.15 nm for the as-prepared film) does not change significantly upon serum exposure under most conditions (see Figure 3b). As the only exception, a significant increase in RMS surface roughness is observed for the 10% serum at pH 8. This behavior most likely reflects the complex interplay between protein adsorption and surface dissolution. However, it should be noted that serum exposure did not lead to any notable corrosion of the iron films, which maintained their mirror finish during all experiments. In order to verify whether serum protein adsorption passivates the surface of the metal films, we also characterized the samples after serum exposure by cyclic voltammetry (CV, see Supplementary Materials). However, the corresponding cyclic voltammograms indicate no inhibition of the electrochemical surface activity due to serum protein adsorption (see Figure S4).

### 2.2. PM-IRRAS Results

**Figure 4:**
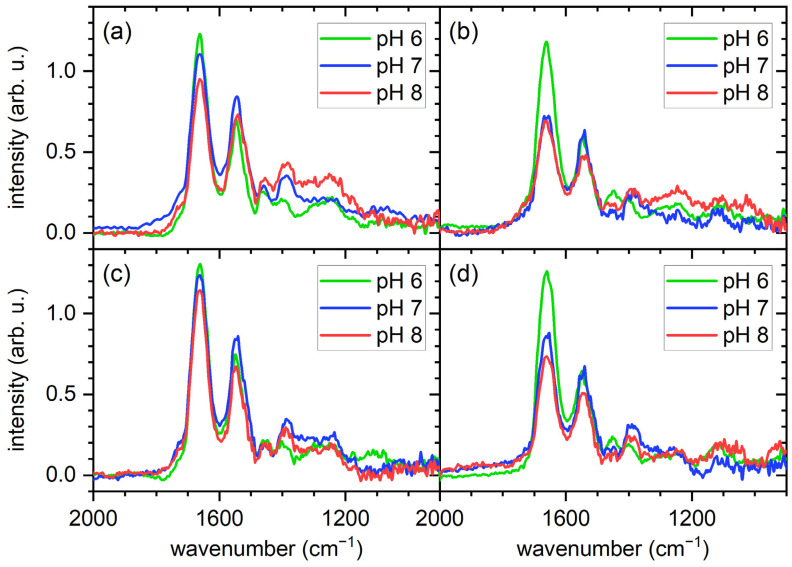
PM-IRRA spectra of the gold (**a**,**c**) and iron (**b**,**d**) films after exposure to 1% (**a**,**b**) and 10% (**c**,**d**) human serum at different pH values, showing the amide I to amide III region.

In order to obtain more quantitative information regarding the total amount of adsorbed proteins and possibly the composition of the protein films, we turned to PM-IRRAS. For this, we focused on the amide I to amide III region, which is composed of IR absorption bands specific to the polypeptide backbone. As can be seen in Figure 4, the amide I and amide II bands at about 1650 cm^−1^ and 1550 cm^−1^, respectively, can be clearly identified in all recorded spectra. The amide III bands, however, are rather weak and barely distinguishable from the background noise.

**Figure 5:**
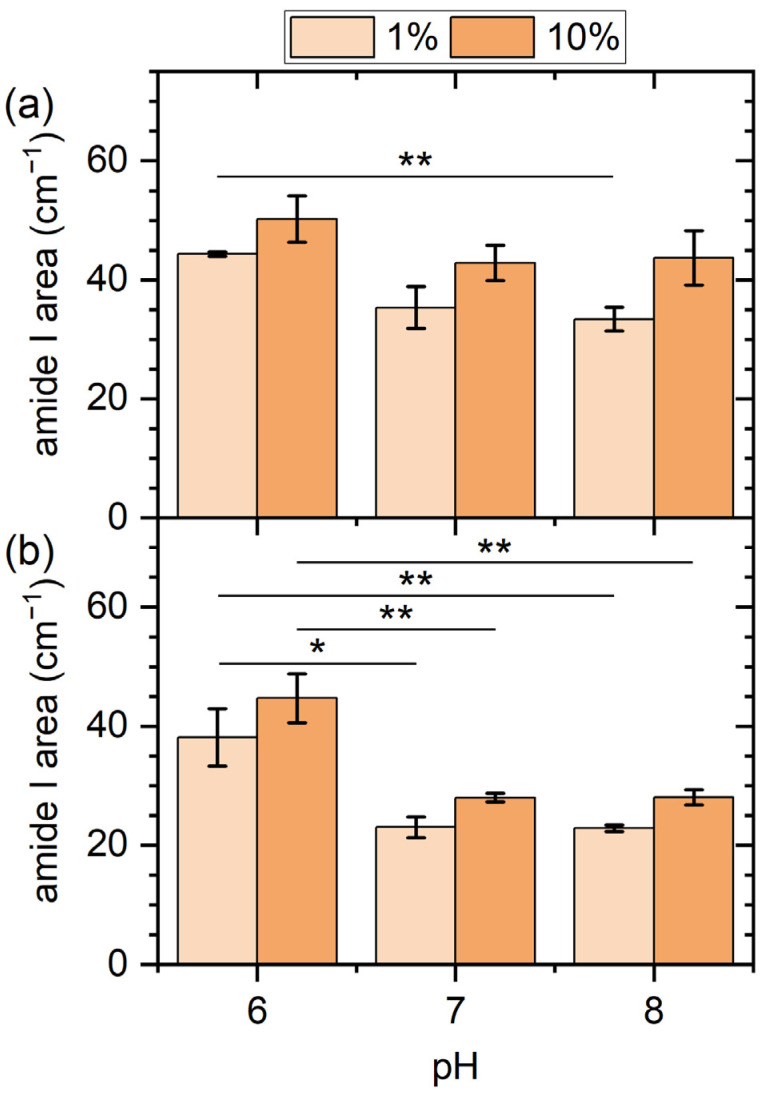
Integrated amide I band area values for the gold (**a**) and iron (**b**) films after exposure to human serum at different concentrations and pH values. Values represent averages of two to four spectra recorded on the surfaces of two to four identically treated samples. Error bars indicate standard deviations. Significances (two-tailed distribution, homoscedastic) are indicated as * (*p* < 0.05), ** (*p* < 0.01), and *** (*p* < 0.001).

The PM-IRRA spectra shown in Figure 4 reveal some interesting dependencies. In particular, the intensities of the amide I bands exhibit rather similar pH dependencies for both surfaces and serum concentrations. In general, it seems that the amide I intensity is increasing with decreasing pH. Since the amide I band is composed mostly of C=O stretching vibrations with a smaller contribution from C-N stretching vibrations, both of which originate in the polypeptide backbone, its integrated intensity is a measure of the total amount of adsorbed proteins [9]. The observed increase in amide I intensity with decreasing pH thus indicates enhanced serum protein adsorption at pH 6 compared to pH 7 and pH 8 for both surfaces. To quantify this effect, the amide I band areas were integrated for all conditions (see Figure 5). Indeed, for all the investigated conditions, pH 6 shows the largest amide I integral, even though the observed trends are not in all cases statistically significant, such as for the gold surface in contact with 10% serum (Figure 5a). Note that we have also measured the thickness of the adsorbed protein films on the gold surface by ellipsometry, which shows a similar pH dependence (see Figure S3).

The apparent pH dependence of the total amount of adsorbed proteins may be explained by considering that at a serum concentration of about 40 mg/mL [15], HSA is the major protein component in human serum (see Table 1). Since HSA has an isoelectric point around 4.7 [26], shifting the pH closer to that value, albeit rather modestly, results in a lower absolute net charge (see Table 2). Considering that protein adsorption in general reaches its maximum when the protein is uncharged [15], it appears reasonable that the adsorption of this already abundant protein is further enhanced at pH 6. For comparison, IgG, which is another abundant plasma protein with a serum concentration around 15 mg/mL [15], has an isoelectric point closer to 7 [27] (see Table 1) and should thus show the inverse trend. This interpretation is further supported by the fact that both surfaces show similar behavior, which suggests that the effect originates in protein properties instead of surface properties. Furthermore, Figure 5 also shows a weak to modest concentration dependence of protein adsorption at both surfaces, with exposure to 10% serum resulting in a ~10 to ~30% increase in the amide I area.

When it comes to surface-specific differences, the data in Figure 5 indicate a larger total amount of adsorbed proteins at the gold surface for all conditions. Increased protein adsorption at the gold compared to other surfaces is a phenomenon frequently observed for many proteins and peptides [24,37,38], including serum albumin [39]. Increased protein adsorption at the gold surface would also provide an explanation for the smoothing of the surface topography revealed by AFM in Figure 2, which was observed only for the gold but not for the iron film. It should be noted, however, that the enhanced intensities of the amide I bands observed for the gold surface may also be due to a larger IR reflection coefficient compared to the iron film.

**Figure 6:**
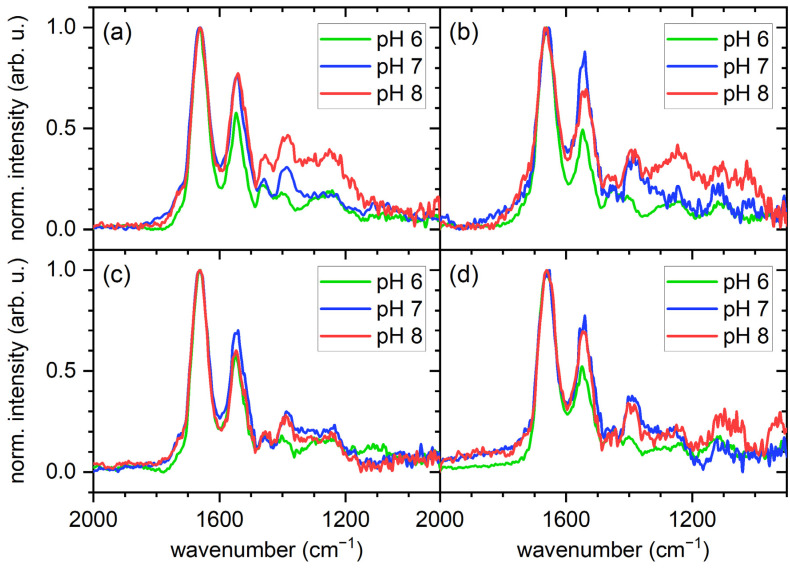
Same spectra of the gold (**a**,**c**) and iron (**b**,**d**) films after exposure to 1% (**a**,**b**) and 10% (**c**,**d**) human serum at different pH values as shown in Figure 4, but normalized to the maximum intensity of the amide I peak.

As discussed above, the pH dependence of the total amount of adsorbed proteins observed in Figure 5 hints at an increased adsorption of HSA at the lower pH value. Since multiprotein adsorption from a complex mixture is considered here, increased adsorption of one or a few protein species will most likely result in a different composition of the adsorbed protein films. Therefore, we next tried to assess such compositional changes by closer examination of the amide I bands. The chemical groups in the polypeptide backbone that contribute to the amide I band participate in the H bonds that stabilize the secondary structure elements of the protein, so that different secondary structure elements can be assigned to different wavenumbers within the amide I region [9]. For a large protein with complex secondary structure, this results in a broad amide I band with a complex shape that can be considered the protein’s structural fingerprint. In order to assess whether a change in the composition of the adsorbed protein film results in a change in the amide I shape, we normalized the spectra shown in Figure 4 with respect to the maximum intensity of the amide I band. As can be seen in Figure 6, the normalized spectra unfortunately do not reveal any clearly identifiable differences in the shapes of the amide I bands obtained at different pH values. This is most likely because the recorded amide I bands are superpositions of many differently shaped amide I bands specific for the large number of protein species present in the adsorbed films. Furthermore, many proteins and in particular serum albumin undergo denaturation during adsorption at solid surfaces, which often is accompanied by drastic changes in the relative occurrence of some secondary structure elements [9].

**Figure 7:**
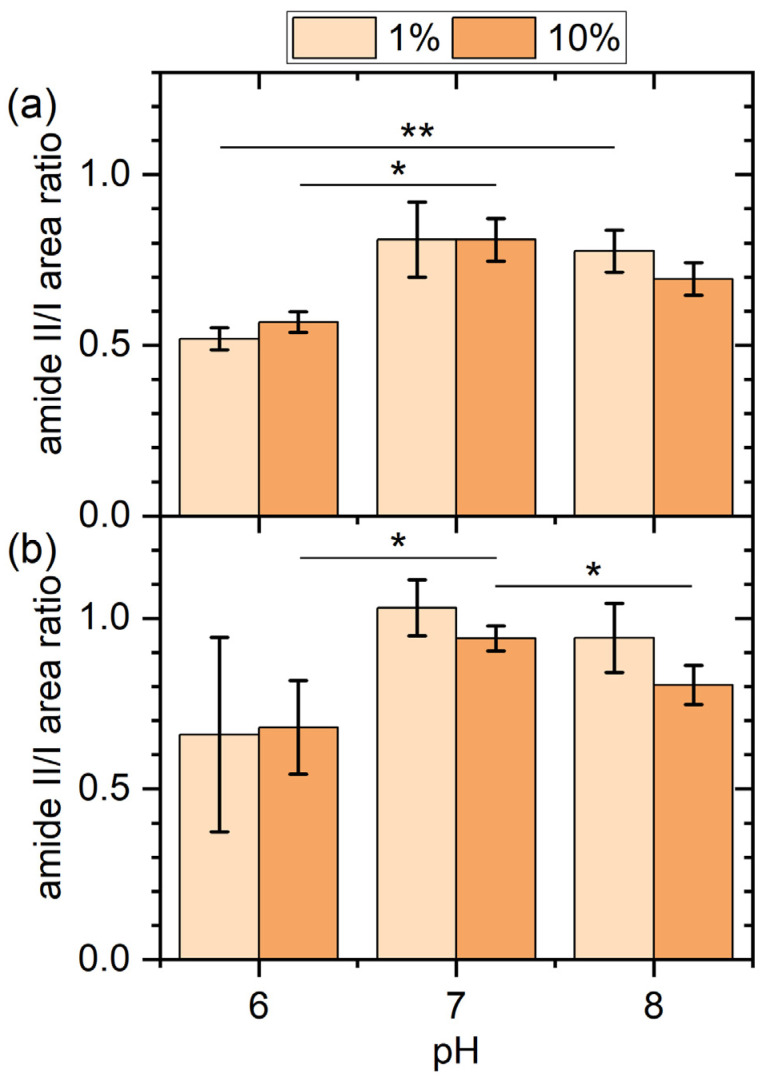
Ratios of the amide II and amide I peak areas for the gold (**a**) and iron (**b**) films after exposure to human serum at different concentrations and pH values. Values represent averages of two to four spectra recorded at different positions on the surfaces of two to four identically treated samples. Error bars indicate standard deviations. Significances (two-tailed distribution, homoscedastic) are indicated as * (*p* < 0.05), ** (*p* < 0.01), and *** (*p* < 0.001).

Even though no changes in the shapes of the amide I bands can be identified in the normalized spectra shown in Figure 6, there are nevertheless some variations in other features that may hint at different film compositions. The first feature is the amide II band. The amide II band is more complex than the amide I band and derives mostly from the in-plane bending vibration of N-H groups with smaller contributions from C-N and C-C stretching vibrations. Therefore, it shows a similar sensitivity to secondary structure as the amide I band. As can be seen in Figure 6 for both surfaces and serum concentrations, changes in pH lead to rather drastic changes in the intensity of the normalized amide II bands, while the normalized amide I bands are barely affected. To quantify this effect, the amide II/I area ratio was calculated for all spectra and is displayed in Figure 7. For both surfaces and serum concentrations, we find a minimum amide II/I area ratio at pH 6, even though the large error bars sometimes result in the differences being non-significant. Two effects may contribute to this decrease in the amide II/I area ratio. The most obvious is a change in the protein composition of the adsorbed film, which would be in line with the above interpretation. However, changes in a protein’s secondary structure can also account for such a change in the amide II/I area ratio [40], since different secondary structure elements may have different absorption coefficients. Such changes in secondary structure may result from both different pH values in bulk solution and differences in adsorption-induced protein denaturation. Therefore, it is not clear whether this change in the amide II/I area ratio is indicative of a change in protein film composition, structure, or both.

The second interesting feature is the weak high-wavenumber shoulder of the amide I band between 1750 and 1700 cm^−1^. IR absorption bands beyond 1700 cm^−1^ are usually attributed to the protonated carboxyl groups located in the side chains of Asp and Glu [9,41]. In all spectra shown in Figure 6, we find that this high-wavenumber shoulder of the amide I band is weaker or even completely absent at pH 6 compared to the higher pH values. This is particularly obvious for the gold surfaces, where the high-pH spectra exhibit a rather well resolved shoulder. For the oxidized iron surface, the effect is less pronounced, which may be attributed to the lower amount of adsorbed proteins that contribute to the overall intensity. The disappearance of the high-wavenumber shoulder at low pH values is rather surprising, since one would expect a lower pH to result in an increase in the number of protonated carboxyl groups and not in a decrease. Even more surprising is the fact that the same trend can also be observed for the weak absorption band around 1400 cm^−1^, which corresponds to the deprotonated carboxylate group [41]. Here, we also observe a decrease in intensity with decreasing pH, suggesting a decrease in the number of deprotonated carboxylate groups. Together, both observations are indicative of a loss of Asp and/or Glu, which can only be the result of a change in the composition of the adsorbed protein films. Since HSA and IgG have a rather similar Asp/Glu content of 10–20 w% [42,43], the observed decrease in Asp/Glu absorption a pH 6 furthermore suggests the involvement of other, less abundant protein species, which show stronger adsorption at higher pH values.

## 3. Materials and Methods

### 3.1. Thin Film Deposition

The thin metal films were deposited on commercial Si(100) wafers (p-doped, Siegert Wafer GmbH, Aachen, Germany) with native surface oxide. After RCA1 cleaning and subsequent rinsing in water and ethanol, a 10 nm Cr (99.95%, Umicore AG & Co. KG, Hanau, Germany) buffer layer followed by 150 nm Au (99.999%, Goodfellow GmbH, Hamburg, Germany) were deposited by thermal evaporation (tectra GmbH, Frankfurt am Main, Germany). In order to provide sufficient reflectivity for PM-IRRAS, the Fe (99.95%, Angstrom Engineering Inc., Cambridge, ON, Canada) films (30 nm) were deposited on top of the Au films with an intermediate 3 nm Cr (99.95%, EVOCHEM Advanced Materials GmbH, Offenbach am Main, Germany) buffer layer by magnetron sputter deposition (Angstrom Engineering Inc., Canada). The chemical composition of the Fe films with native surface oxide was characterized by X-ray photoelectron spectroscopy (XPS, see Supplementary Materials). Right before each adsorption experiment, the sample surfaces were rinsed with ethanol and dried in a stream of nitrogen.

### 3.2. Serum Exposure

Heat-inactivated human serum from male AB clotted whole blood (product number H5667, Sigma-Aldrich, St. Louis, MO, USA) was diluted in aerated phosphate buffered saline (PBS, VWR International GmbH, Darmstadt, Germany) to obtain the desired concentration (1% or 10%). The pH of the PBS was adjusted between pH 6 and pH 8 using HCl (35%) and NaOH (>99%). Serum dilutions were prepared fresh each day to ensure comparability of the results. The different serum samples (100 µL) were deposited on the different sample surfaces (1 × 1 cm^2^) and incubated for 2 h at room temperature. After incubation, the sample surfaces were rinsed with ca. 3 mL HPLC-grade water (Carl Roth GmbH + Co. KG, Karlsruhe, Germany) and dried in a stream of ultra-pure air.

### 3.3. AFM

The surface topography of the dry samples was analyzed by AFM using an Agilent 5500 system (Agilent Technologies, Inc., Santa Clara, CA, USA) operated in intermittent contact mode in air with MikroMasch NSC18/AIBS cantilevers (NanoAndMore GmbH, Wetzlar, Germany). Images were recorded at a scan size and resolution of 3 × 3 µm^2^ and 512 × 512 px, respectively. The images were analyzed using Gwyddion 2.60 open-source software [44].

### 3.4. PM-IRRAS

PM-IRRAS was performed using a Bruker Vertex 70 spectrometer with a photoelastic ZnSe modulator (PMA50, Bruker Corporation, Billerica, MA, USA). The angle of incidence was 80° with respect to the surface normal. The reflected light was detected using a liquid-nitrogen-cooled mercury cadmium telluride (MCT) detector (Bruker Corporation, USA). The recorded spectra were processed and analyzed using OPUS 5.5 (Bruker Corporation, USA). For determining the amide I and amide II band areas, the integral between the respective bands and a straight line drawn between the intensity values of two specified frequencies (amide I: 1700–1600 cm^−1^; amide II: 1600–1480 cm^−1^) was calculated. This was performed using the original spectra without background correction.

## 4. Conclusions

In summary, we investigated the pH dependence of multiprotein adsorption at gold and oxidized iron surfaces exposed to human serum using AFM and PM-IRRAS. AFM allowed us to quantify adsorption-induced changes in surface topography, while PM-IRRAS was used to quantify the total amount of adsorbed proteins and to qualitatively assess compositional changes in the adsorbed protein films. AFM revealed that on average, serum protein adsorption at the gold surface resulted in a smoother surface morphology, without any pronounced dependencies on pH or serum concentration. For the iron surface, however, no statistically significant changes in morphology due to protein adsorption were observed. PM-IRRAS showed a pH dependence of the total amount of adsorbed proteins for both surfaces, which was even more pronounced than the dependence on serum concentration. Finally, close inspection of the amide II region of the PM-IRRA spectra and in particular the absorption bands of the amino acid side chains revealed clear differences in the composition of the adsorbed multiprotein films at pH 6 compared to pH 7 and pH 8. Since the observed pH-dependent effects are rather similar for both the gold and the oxidized iron surface, we suspect that they are not primarily governed by surface properties but rather by the pH sensitivity of the adsorbing proteins. Because of its isoelectric point of about 4.7 and its high abundance in serum, we assume that protein adsorption at pH 6 is mostly driven by HSA, which replaces other proteins with lower surface affinities in the multiprotein film. Our results demonstrate the potential of PM-IRRAS not only to detect variations in the total amount of proteins adsorbed at metallic surfaces from complex mixtures such as serum but also to assess compositional changes in the adsorbed multiprotein films, albeit only at an indirect and qualitative level. Furthermore, if thin films deposited on top of sufficiently thick gold films are used as substrates for adsorption, this technique can also be extended to non-metallic surfaces such as oxides [45].

## Figures and Tables

**Table 1:** Most abundant proteins in human serum [15].

Protein	Concentration (mg/mL)	Relative Portion (%)	Molecular Weight (kDa)	Isoelectric Point
Albumin	40	50–67	66	4.7 [26]
IgG	15	19–25	150	6.2–8.6 [27]
α_1_-Antitrypsin	3	4–5	54	5.1 [28]
Low-density lipoprotein	3	4–5	5000	5.1–5.8 [29]
α_2_-Macroglobulin	3	4–5	725	5.3 [30]
Transferrin	2.6	3–4	77	5.2–5.6 [31]
IgA	2.3	3–4	162	4.5–6.8 [32]
α_2_-Haptoglobins	2	3–4	100	5.55–6.52 [33]
High-density lipoprotein	2	3–4	195	3.8–7.4 [34]
Complement 3	1.6	2–3	180	5.75 [35]

**Table 2:** Calculated net charge of HSA at the different pH values used in the present study [36].

pH	Net Charge
6	−0.6
7	−12.2
8	−24.2

## Data Availability

The data presented in this study are available on request from the corresponding author.

## Supplementary Materials

The following supporting information can be downloaded at: https://www.mdpi.com/article/10.3390/molecules28166060/s1, Figure S1: XPS survey spectrum of the deposited iron film; Figure S2: High-resolution XPS spectra of Fe 2p_3/2_ and O 1s; Figure S3: Thickness of the protein films adsorbed at the gold surfaces after exposure to human serum at different concentrations and pH values; Figure S4: Cyclic voltammograms of the iron and gold films in PBS after exposure to 1% and 10% human serum; Table S1: Results of the XPS elemental analysis of the deposited iron film.

## References

[1] Jain A. (2015). Body fluid composition. Pediatr. Rev..

[2] Wilson C.J., Clegg R.E., Leavesley D.I., Pearcy M.J. (2005). Mediation of biomaterial-cell interactions by adsorbed proteins: A review. Tissue Eng..

[3] Yang Y., Yu M., Böke F., Qin Q., Hübner R., Knust S., Schwiderek S., Grundmeier G., Fischer H., Keller A. (2021). Effect of nanoscale surface topography on the adsorption of globular proteins. Appl. Surf. Sci..

[4] Yang Y., Knust S., Schwiderek S., Qin Q., Yun Q., Grundmeier G., Keller A. (2021). Protein Adsorption at Nanorough Titanium Oxide Surfaces: The Importance of Surface Statistical Parameters beyond Surface Roughness. Nanomaterials.

[5] Hemmersam A.G., Rechendorff K., Foss M., Sutherland D.S., Besenbacher F. (2008). Fibronectin adsorption on gold, Ti-, and Ta-oxide investigated by QCM-D and RSA modelling. J. Colloid Interface Sci..

[6] Höök F., Vörös J., Rodahl M., Kurrat R., Böni P., Ramsden J., Textor M., Spencer N., Tengvall P., Gold J. (2002). A comparative study of protein adsorption on titanium oxide surfaces using in situ ellipsometry, optical waveguide lightmode spectroscopy, and quartz crystal microbalance/dissipation. Colloids Surf. B Biointerfaces.

[7] Keller T.F., Schönfelder J., Reichert J., Tuccitto N., Licciardello A., Messina G.M.L., Marletta G., Jandt K.D. (2011). How the surface nanostructure of polyethylene affects protein assembly and orientation. ACS Nano.

[8] Nguyen D.H.K., Pham V.T.H., Al Kobaisi M., Bhadra C., Orlowska A., Ghanaati S., Manzi B.M., Baulin V.A., Joudkazis S., Kingshott P. (2016). Adsorption of Human Plasma Albumin and Fibronectin onto Nanostructured Black Silicon Surfaces. Langmuir.

[9] Desroches M.J., Chaudhary N., Omanovic S. (2007). PM-IRRAS investigation of the interaction of serum albumin and fibrinogen with a biomedical-grade stainless steel 316LVM surface. Biomacromolecules.

[10] Bergkvist M., Carlsson J., Oscarsson S. (2003). Surface-dependent conformations of human plasma fibronectin adsorbed to silica, mica, and hydrophobic surfaces, studied with use of Atomic Force Microscopy. J. Biomed. Mater. Res. A.

[11] Firkowska-Boden I., Helbing C., Dauben T.J., Pieper M., Jandt K.D. (2020). How Nanotopography-Induced Conformational Changes of Fibrinogen Affect Platelet Adhesion and Activation. Langmuir.

[12] Pasche S., Vörös J., Griesser H.J., Spencer N.D., Textor M. (2005). Effects of ionic strength and surface charge on protein adsorption at PEGylated surfaces. J. Phys. Chem. B.

[13] Wasilewska M., Adamczyk Z., Pomorska A., Nattich-Rak M., Sadowska M. (2019). Human Serum Albumin Adsorption Kinetics on Silica: Influence of Protein Solution Stability. Langmuir.

[14] Huang L., Shao D., Wang Y., Cui X., Li Y., Chen Q., Cui J. (2021). Human body-fluid proteome: Quantitative profiling and computational prediction. Brief. Bioinform..

[15] Dee K.C., Puleo D.A., Bizios R. (2002). An Introduction to Tissue-Biomaterial Interactions.

[16] Benesch J., Svedhem S., Svensson S.C., Valiokas R., Liedberg B., Tengvall P. (2001). Protein adsorption to oligo(ethylene glycol) self-assembled monolayers: Experiments with fibrinogen, heparinized plasma, and serum. J. Biomater. Sci. Polym. Ed..

[17] Ladd J., Zhang Z., Chen S., Hower J.C., Jiang S. (2008). Zwitterionic polymers exhibiting high resistance to nonspecific protein adsorption from human serum and plasma. Biomacromolecules.

[18] Lestelius M., Liedberg B., Tengvall P. (1997). In Vitro Plasma Protein Adsorption on ω-Functionalized Alkanethiolate Self-Assembled Monolayers. Langmuir.

[19] Yang W., Xue H., Li W., Zhang J., Jiang S. (2009). Pursuing “zero” protein adsorption of poly(carboxybetaine) from undiluted blood serum and plasma. Langmuir.

[20] Yang W., Chen S., Cheng G., Vaisocherová H., Xue H., Li W., Zhang J., Jiang S. (2008). Film thickness dependence of protein adsorption from blood serum and plasma onto poly(sulfobetaine)-grafted surfaces. Langmuir.

[21] Tosatti S., Paul S., Askendal A., VandeVondele S., Hubbell J.A., Tengvall P., Textor M. (2003). Peptide functionalized poly(l-lysine)-g-poly(ethylene glycol) on titanium: Resistance to protein adsorption in full heparinized human blood plasma. Biomaterials.

[22] Rosengren Å., Pavlovic E., Oscarsson S., Krajewski A., Ravaglioli A., Piancastelli A. (2002). Plasma protein adsorption pattern on characterized ceramic biomaterials. Biomaterials.

[23] Boyd A.R., Burke G.A., Duffy H., Holmberg M., O’ Kane C., Meenan B.J., Kingshott P. (2011). Sputter deposited bioceramic coatings: Surface characterisation and initial protein adsorption studies using surface-MALDI-MS. J. Mater. Sci. Mater. Med..

[24] Hemmersam A.G., Foss M., Chevallier J., Besenbacher F. (2005). Adsorption of fibrinogen on tantalum oxide, titanium oxide and gold studied by the QCM-D technique. Colloids Surf. B Biointerfaces.

[25] Huang J., Gonzalez Orive A., Krüger J.T., Hoyer K.-P., Keller A., Grundmeier G. (2022). Influence of proteins on the corrosion of a conventional and selective laser beam melted FeMn alloy in physiological electrolytes. Corros. Sci..

[26] Vlasova I.M., Saletsky A.M. (2009). Study of the denaturation of human serum albumin by sodium dodecyl sulfate using the intrinsic fluorescence of albumin. J. Appl. Spectrosc..

[27] Yang D., Kroe-Barrett R., Singh S., Laue T. (2019). IgG Charge: Practical and Biological Implications. Antibodies.

[28] Viglio S., Iadarola P., D’Amato M., Stolk J. (2020). Methods of Purification and Application Procedures of Alpha1 Antitrypsin: A Long-Lasting History. Molecules.

[29] Satchell L., Leake D.S. (2012). Oxidation of low-density lipoprotein by iron at lysosomal pH: Implications for atherosclerosis. Biochemistry.

[30] Back S.A., Alhadeff J.A. (1983). Differential isoelectric focusing properties of crude and purified human alpha 2-macroglobulin and alpha 2-macroglobulin-proteinase complexes. J. Chromatogr..

[31] Hovanessian A.G., Awdeh Z.L. (1976). Gel isoelectric focusing of human-serum transferrin. Eur. J. Biochem..

[32] Monteiro R.C., Halbwachs-Mecarelli L., Roque-Barreira M.C., Noel L.H., Berger J., Lesavre P. (1985). Charge and size of mesangial IgA in IgA nephropathy. Kidney Int..

[33] Shibata K., Constans J., Viau M., Matsumoto H. (1982). Polymorphism of the haptoglobin peptides by isoelectric focusing electrophoresis and isoelectric point determinations. Hum. Genet..

[34] Sundaram G.S., Mackenzie S.L., Sodhi H.S. (1974). Preparative isoelectric focusing of human serum high-density lipoprotein (HDL3). Biochim. Biophys. Acta.

[35] Nelson R.A., Brebner E. (1974). Isoelectric focusing of components of the complement system and certain related proteins in human serum. Immunol. Commun..

[36] Protein Calculator v3.4. http://protcalc.sourceforge.net/.

[37] Dolatshahi-Pirouz A., Kolman N., Arpanaei A., Jensen T., Foss M., Chevallier J., Kingshott P., Baas J., Søballe K., Besenbacher F. (2011). The adsorption characteristics of osteopontin on hydroxyapatite and gold. Mater. Sci. Eng. C.

[38] Yang Y., Schwiderek S., Grundmeier G., Keller A. (2021). Strain-Dependent Adsorption of Pseudomonas aeruginosa-Derived Adhesin-Like Peptides at Abiotic Surfaces. Micro.

[39] Tidwell C.D., Castner D.G., Golledge S.L., Ratner B.D., Meyer K., Hagenhoff B., Benninghoven A. (2001). Static time-of-flight secondary ion mass spectrometry and x-ray photoelectron spectroscopy characterization of adsorbed albumin and fibronectin films. Surf. Interface Anal..

[40] Ishida K.P., Griffiths P.R. (1993). Comparison of the Amide I/II Intensity Ratio of Solution and Solid-State Proteins Sampled by Transmission, Attenuated Total Reflectance, and Diffuse Reflectance Spectrometry. Appl. Spectrosc..

[41] Barth A. (2000). The infrared absorption of amino acid side chains. Prog. Biophys. Mol. Biol..

[42] Belinskaia D.A., Voronina P.A., Batalova A.A., Goncharov N.V. (2021). Serum Albumin. Encyclopedia.

[43] Shaw A.L., Mathews D.W., Hinkle J.E., Petschow B.W., Weaver E.M., Detzel C.J., Klein G.L., Bradshaw T.P. (2016). Absorption and safety of serum-derived bovine immunoglobulin/protein isolate in healthy adults. Clin. Exp. Gastroenterol..

[44] Nečas D., Klapetek P. (2012). Gwyddion: An open-source software for SPM data analysis. Open Phys..

[45] Torun B., Giner I., Grundmeier G., Ozcan O. (2017). In situ PM-IRRAS studies of organothiols and organosilane monolayers-ZnO interfaces at high water activities. Surf. Interface Anal..

