# Supplementary material for: Multiprotein Adsorption from Human Serum at Gold and Oxidized Iron Surfaces Studied by Atomic Force Microscopy and Polarization-Modulation Infrared Reflection Absorption Spectroscopy
Source: Molecules. 2023 Aug 15;28(16):6060. doi: 10.3390/molecules28166060 (PMC10459451; doi:10.3390/molecules28166060)
Supplement: Supplementary file 1 [file molecules-28-06060-s001.zip › molecules-2542469-supplementary.pdf]

Supplementary Materials

Multiprotein Adsorption from Human Serum at Gold and Oxidized Iron Surfaces Studied by Atomic Force Microscopy and Polarization-Modulation Infrared Reflection Absorption Spectroscopy

Jingyuan Huang, Yunshu Qiu, Felix Lücke, Jiangling Su, Guido Grundmeier, Adrian Keller

X-ray photoelectron spectroscopy (XPS)

X-ray photoelectron spectroscopy (XPS) measurements of the deposited iron film were performed with an Omicron ESCA+ system (Omicron NanoTechnology, Taunusstein, Germany) equipped with a monochromatic Al-Kα X-ray source (1486.7 eV) with a spot diameter of 600 µm and a take-off angle of 30°, coupled to a hemispheric analyzer, at a chamber pressure of $< 5 \times 10^{-10}$ mbar. Survey spectra and high-resolution spectra were recorded using a pass energy of 100 eV and 20 eV, respectively. All spectra were calibrated using the C 1s peak (284.6 eV) as the internal reference. The software CASA XPS (Casa Software Ltd., Wilmslow, UK) was used for the peak fitting and data analysis. A Shirley background was chosen for peak fitting. The peaks were fitted using a peak shape consisting of a convolution of a Gaussian (30%) and Lorentzian (70%) shape. For the quantification, relative sensitivity factors supplied from Omicron GmbH were implemented in the CASA XPS database (Casa Software Ltd., Wilmslow, UK).

The survey spectrum of the iron film as shown in the Figure S1 confirms the presence of C, O, Fe on the surface. The atomic percentage composition of iron film is summarized in the Table S1. Figure S2 shows the high-resolution XPS spectra in the regions of Fe $2p_{3/2}$ (a) and O 1s (b), respectively. The high-resolution Fe $2p_{3/2}$ spectrum shown in Figure S2a could be fitted with three components, i.e., the peaks of Fe $2p_{3/2}$ at 710.0 eV, 711.1 eV, and 707.4 eV which are attributed to Fe(II), Fe(III), and Fe(metal), respectively. The high-resolution O 1s spectrum in the Figure S2b shows two components. The peak at 529.7eV is attributed

to iron oxides, the one at 530.9 eV is attributed to the bulk oxygen and surface adsorbed oxygen, and the one at 531.6 eV is attributed to the FeOOH. In summary, the XPS characterization suggests the formation of a mixed oxide layer consisting of Fe_2O_3, Fe_3O_4 and FeOOH on the deposited iron films.

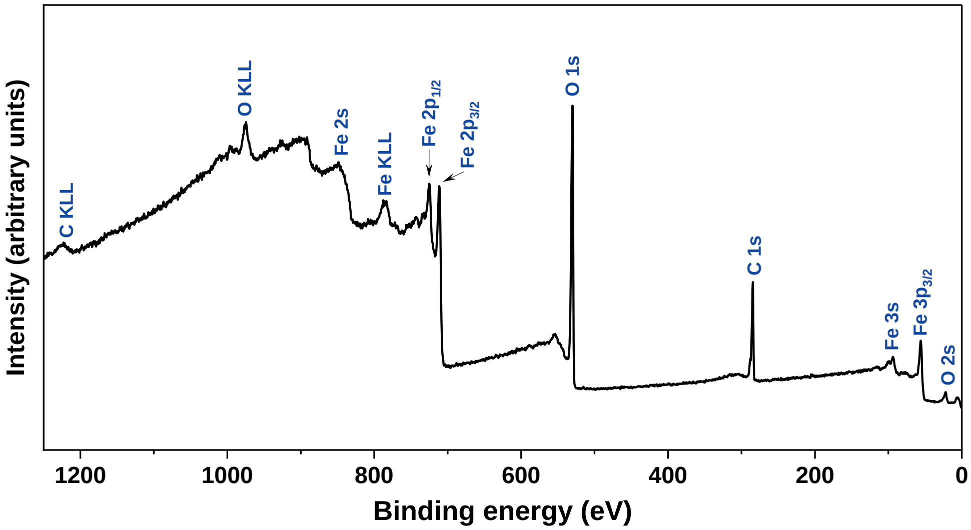

Figure S1. XPS survey spectrum of the deposited iron film.

Table S1. Results of the XPS elemental analysis of the deposited iron film.

**Element**	**C 1s**	**O 1s**	**Fe $2p_{3/2}$**
Concentration (at%)	43.5	47.3	9.2

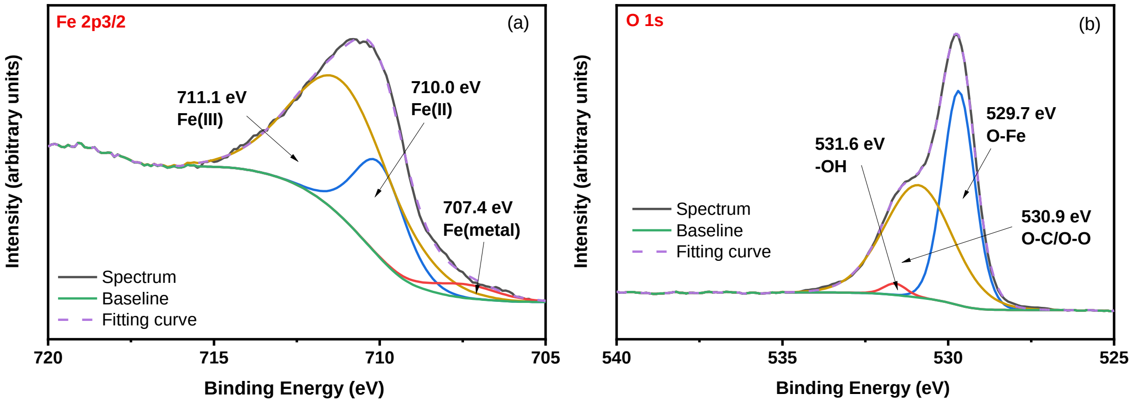

Figure S2. High-resolution XPS spectra of Fe $2p_{3/2}$ (a) and O 1s (b).

Ellipsometry

The thickness of the adsorbed protein layers at the gold surfaces was determined by ellipsometry (auto nulling ellipsometer, Ep3, Accurion GmbH, Göttingen, Germany). Two-zone measurements were performed in the wavelength range from 363.7 nm to 550.1 nm at an incidence angle of 70°, and three points were measured for each sample. A blank gold substrate without adsorbed proteins was also measured. A model of the blank substrate and the adsorbed protein layer was constructed, and the data were fitted in the EP4 software using a Cauchy dispersion function

$$n(\lambda) = A + \frac{B}{\lambda^2}$$

with the refractive index *n*, the Cauchy parameters *A* and *B*, and the wavelength λ. The so determined thickness values are shown in Figure S3.

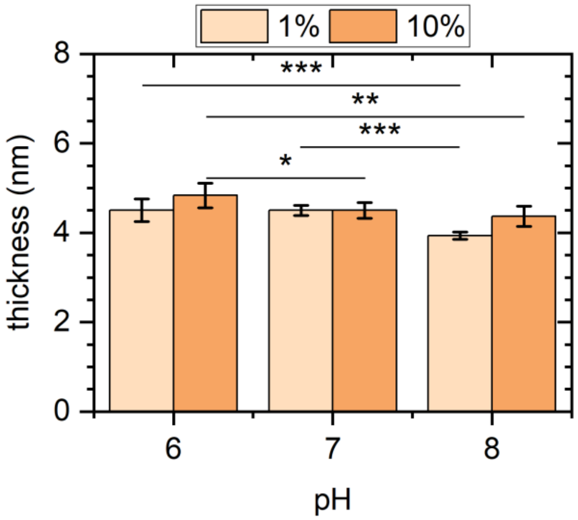

Figure S3. Thickness of the protein films adsorbed at the gold surfaces after exposure to human serum at different concentrations and pH values. Values represent averages of six measurements recorded on the surfaces of two identically treated samples. Error bars indicate standard deviations. Significances (two-tailed distribution, homoscedastic) are indicated as * (p < 0.05), ** (p < 0.01), and *** (p < 0.001).

Cyclic voltammetry (CV)

CV experiments were performed on the gold and iron films after protein adsorption in a three-electrode cell using a Reference 600 potentiostat (Gamry Instruments). The working electrode was the sample, the counter electrode was a gold wire, and the reference electrode was a standard Ag/AgCl electrode (sat. 3 M KCl). The cyclic voltammograms were recorded in PBS between 0.25 and -0.8 V (relative to Ag/AgCl) with a scan rate of 50 mV s^{-1}. Three cycles were recorded each time, using the first cycle for comparison. As can be seen in the corresponding cyclic voltammograms shown in Figure S4, there is rather an increase than a decrease of the peak areas after protein adsorption. Furthermore, while the Fe oxidation/reduction cycle is visible for the Fe substrates, we do not observe a superimposition of the reduction peaks on Au and on Fe. Therefore, CV indicates no inhibition of the electrochemical surface activity due to serum protein adsorption.

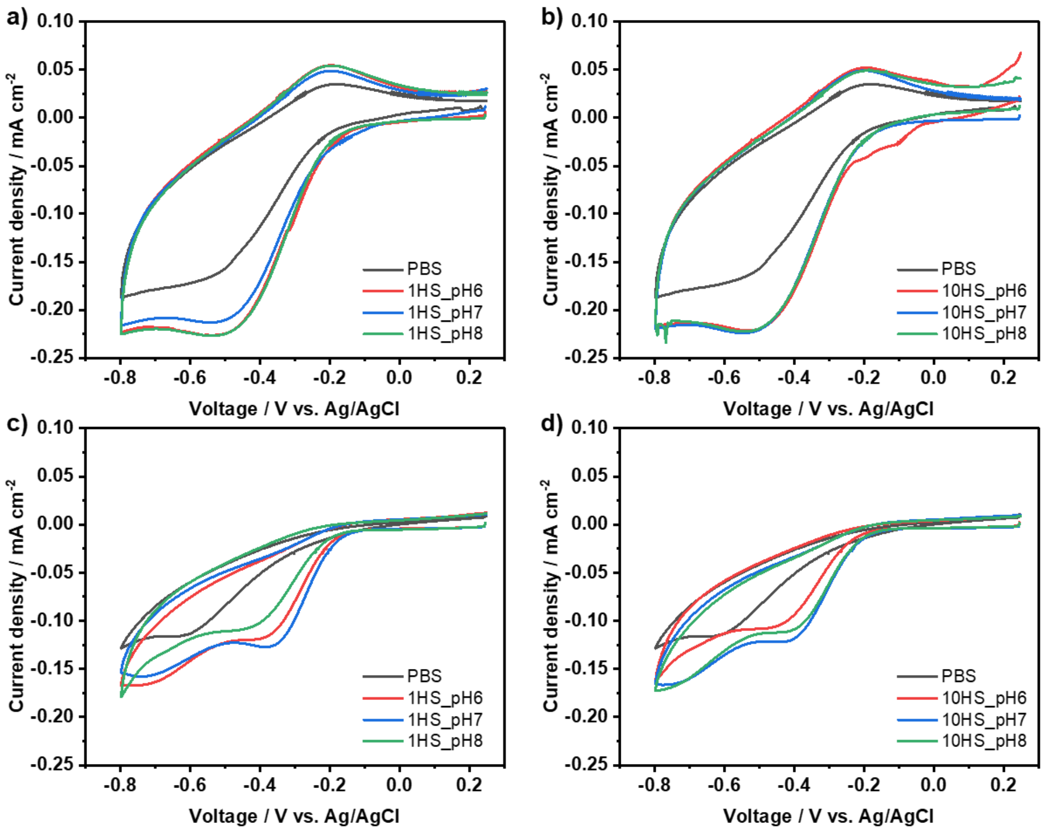

Figure S4. Cyclic voltammograms of the iron (a,b) and gold (c,d) films in PBS after exposure to 1% (a,c) and 10% (b,d) human serum. The curves shown in the plots always represent the first cycle.
